# NLRP3 inflammasome-mediated microglial pyroptosis is critically involved in the development of post-cardiac arrest brain injury

**DOI:** 10.1186/s12974-020-01879-1

**Published:** 2020-07-23

**Authors:** Yuan Chang, Juan Zhu, Di Wang, Hua Li, Yihua He, Kewei Liu, Xiaoqiang Wang, Yuqin Peng, Suyue Pan, Kaibin Huang

**Affiliations:** 1 Department of Neurology, Nanfang Hospital, Southern Medical Univerisity, North Avenue 1838#, Guangzhou, Guangzhou, 510515 China; 2 Department of Dermatology, Zhujiang Hospital, Southern Medical University, Guangzhou, Guangdong China; 3 Department of Neurology, Zhuhai Hospital of Integrated Traditional Chinese and Western Medicine, Zhuhai, China

**Keywords:** Cardiac arrest, Caspase-1, Microglia, NLRP3, Pyroptosis

## Abstract

**Background:**

Brain injury is the leading cause of death and disability in survivors of cardiac arrest, where neuroinflammation is believed to play a pivotal role, but the underlying mechanism remains unclear. Pyroptosis is a pro-inflammatory form of programmed cell death that triggers inflammatory response upon infection or other stimuli. This study aims to understand the role of microglial pyroptosis in post-cardiac arrest brain injury.

**Methods:**

Sprague-Dawley male rats underwent 10-min asphyxial cardiac arrest and cardiopulmonary resuscitation or sham-operation. Flow cytometry analysis, Western blotting, quantitative real-time polymerase chain reaction (qRT-PCR), co-immunoprecipitation, and immunofluorescence were used to evaluate activated microglia and CD11b-positive leukocytes after cardiac arrest and assess inflammasome activation and pyroptosis of specific cellular populations. To further explore the underlying mechanism, MCC950 or Ac-YVAD-cmk was administered to block nod-like receptor family protein 3 (NLRP3) or caspase-1, respectively.

**Results:**

Our results showed that, in a rat model, successful resuscitation from cardiac arrest resulted in microglial pyroptosis and consequential inflammatory infiltration which was mediated by the activation of NLRP3 inflammasome. Targeting NLRP3 and caspase-1, the executor of pyroptosis, with selective inhibitors MCC950 and Ac-YVAD-cmk treatment significantly prevented microglial pyroptosis, reduced infiltration of leukocytes, improved neurologic outcome, and alleviated neuro-pathological damages after cardiac arrest in modeling rats.

**Conclusions:**

This study demonstrates that microglial pyroptosis mediated by NLRP3 inflammasome is critically involved in the pathogenesis of post-cardiac arrest brain injury and provides a new therapeutic strategy.

## Introduction

Sudden cardiac arrest is a leading cause of death and disability in China and worldwide [1, 2]. Despite the unremitting efforts by health personnel and emergency medical services, as well as increasing awareness about cardiopulmonary resuscitation, only 26% of adults successfully resuscitated from out-of-hospital cardiac arrest survived to discharge in China [1]. The main reason for the poor prognosis of patients with successful resuscitation is the post-cardiac arrest syndrome, in which brain injury plays a leading role [3]. Although the mechanism underlying post-cardiac arrest brain injury is not fully understood, neuroinflammation has been widely recognized for playing an essential role, as neuroinflammation is characterized by activation of glial cells, an influx of peripheral immune and inflammatory cells, and release of proinflammatory mediators, including cytokines and adhesion molecules [4]. Microglia, as innate immune cells in the brain, may get overactivated to play an initiative role in these inflammatory cascades [4]. Nevertheless, how microglia are primed to fuel the inflammatory response after cardiac arrest remains largely unknown.

Pyroptosis, an emerging form of programmed necrosis, is driven by many cytosolic pattern recognition receptors represented by the nod-like receptor family protein 3 (NLRP3) [5]. Pyroptosis is inherently inflammatory, and it features rapid plasma-membrane rupture and release of pro-inflammatory intracellular contents such as interleukin 1β (IL-1β) and interleukin 18 (IL-18) [6]. Caspase-1 is the canonical executor of pyroptosis and is also responsible for the processing of gasdermin D (GSDMD), precursors of IL-1β (pro-IL-1β) and IL-18 (pro-IL-18). Furthermore, it should be noted that cleavage of GSDMD by the inflammatory caspases including caspase-1 critically drives pyroptosis by releasing the cleaved gasdermin-N fragment that acts as an intrinsic pyroptosis-inducing factor [7]. Previous studies have demonstrated that NLRP3 is highly expressed in microglia and participates in the assembly of inflammasome by recruiting apoptosis-associated speck-like proteins containing a caspase recruitment domain (ASC) and precursor of caspase-1 (pro-caspase-1), followed by the self-activation of caspase-1 to trigger cell pyroptosis and consequential excessive inflammation [8, 9]. The activation of NLRP3 inflammasome has been reported to involve in the pathophysiology of various neurological disorders, such as ischemic stroke [10], intracerebral hemorrhage [11, 12], and traumatic brain injury [13]. However, after cardiac arrest and cardiopulmonary resuscitation, whether the assembly of NLRP3 inflammasome primes microglia and mediates its pyroptosis and consequential neuroinflammation remains unclear. In addition, whether blocking NLRP3 inflammasome is beneficial for alleviating the post-cardiac arrest brain injury remains to be further explored, although it has been shown that blocking the inflammasome is beneficial in cerebral ischemic diseases like stroke in the previous studies [14].

In this study, by using a rat model of asphyxial cardiac arrest and cardiopulmonary resuscitation, we demonstrate that NLRP3 inflammasome-mediated microglial pyroptosis is critically involved in the development of post-cardiac arrest brain injury. Our data showed that cardiac arrest induced microglial pyroptosis and consequential neuroinflammation, mediated by the activation of NLRP3 inflammasome. Targeting NLRP3 and caspase-1 with two highly selective inhibitors MCC950 and Ac-YVAD-cmk dramatically reduced the number of microglia undergoing pyroptosis, the number of infiltrating leukocytes, the elevated level of IL-1β and IL-18, improved neurologic outcome, and alleviated neuronal injury in cardiac arrest-modeling rats. These results may increase our understanding of the mechanism underlying NLRP3 inflammasome-mediated microglia pyroptosis and the therapeutic effect of NLRP3 and caspase-1 inhibitors in the management of post-cardiac arrest brain injury.

## Materials and methods

### Animals

All animal experiments in this study were approved by the Animal Care and Use Committee of the Nanfang Hospital, Southern Medical University (Guangzhou, China), and adhered to National Institute of Health Guide for the care and use of laboratory animals. Male adult Sprague-Dawley rats (350–400 g) were obtained from the Experimental Animal Center of Southern Medical University and housed in the specific pathogen-free facility at the university under a strict 12-h light/dark cycle with free access to food and water. All efforts were made to minimize the number of animals used in the current study including its suffering.

### Rat model of asphyxial cardiac arrest

The 10-min asphyxial cardiac arrest and cardiopulmonary resuscitation model was performed as described in our previous study [15]. In brief, rats were anesthetized with isoflurane blended with room air (4% for induction and 2% for maintenance; RWD, Shenzhen, China), orotracheally intubated with a 14G cannula (BD, Suzhou, China), and connected to a ventilator (RWD). Intravascular catheters (PE50; Smiths Medical, Ashford, UK) were inserted into the right femoral artery and vein for dynamic blood pressure monitoring and drug delivery, respectively. After 10-min of stabilization, rats were chemically paralyzed by IV Vecuronium (2 mg/kg), and then the ventilator was disconnected for 10 min, typically leading to circulatory arrest in 5 min, which was characterized by cessation of arterial pulse and a decrease in mean arterial pressure (MAP) below 20 mmHg. At the end of 10-min asphyxia, cardiopulmonary resuscitation was initiated by effective ventilation with 100% oxygen as the inspired gas and intravenous administration of epinephrine (0.01 mg/kg). Concurrently, finger chest compressions were conducted by the operator at a rate of about 200 compressions per minute. Additional doses of epinephrine (0.02 mg/kg) were given at 2-min intervals until return of spontaneous circulation (ROSC) was achieved. ROSC was defined as an increase in MAP beyond 60 mmHg lasting at least 10 min. Rats that failed to ROSC within 5 min or were unable to be weaned from ventilator after 1-h observation were excluded from the continuing experiments.

During the whole procedure, core temperature was monitored by a rectal temperature probe (RC-4; Elitech, Xuzhou, China) and maintained at 37.0 ± 0.5 °C with a temperature feedback system (RWD). After spontaneous respiration recovery, rats were weaned from ventilator and extubated. Afterward, all catheters were removed and surgical wounds were sutured. At the end of each experimental period, rats were returned to their cages with easily accessible food and water and were observed in a room of constant temperature (22 °C). Saline (20 mL/kg) was injected daily to rats until they were able to feed themselves.

### Study protocol

**Fig. 1:**
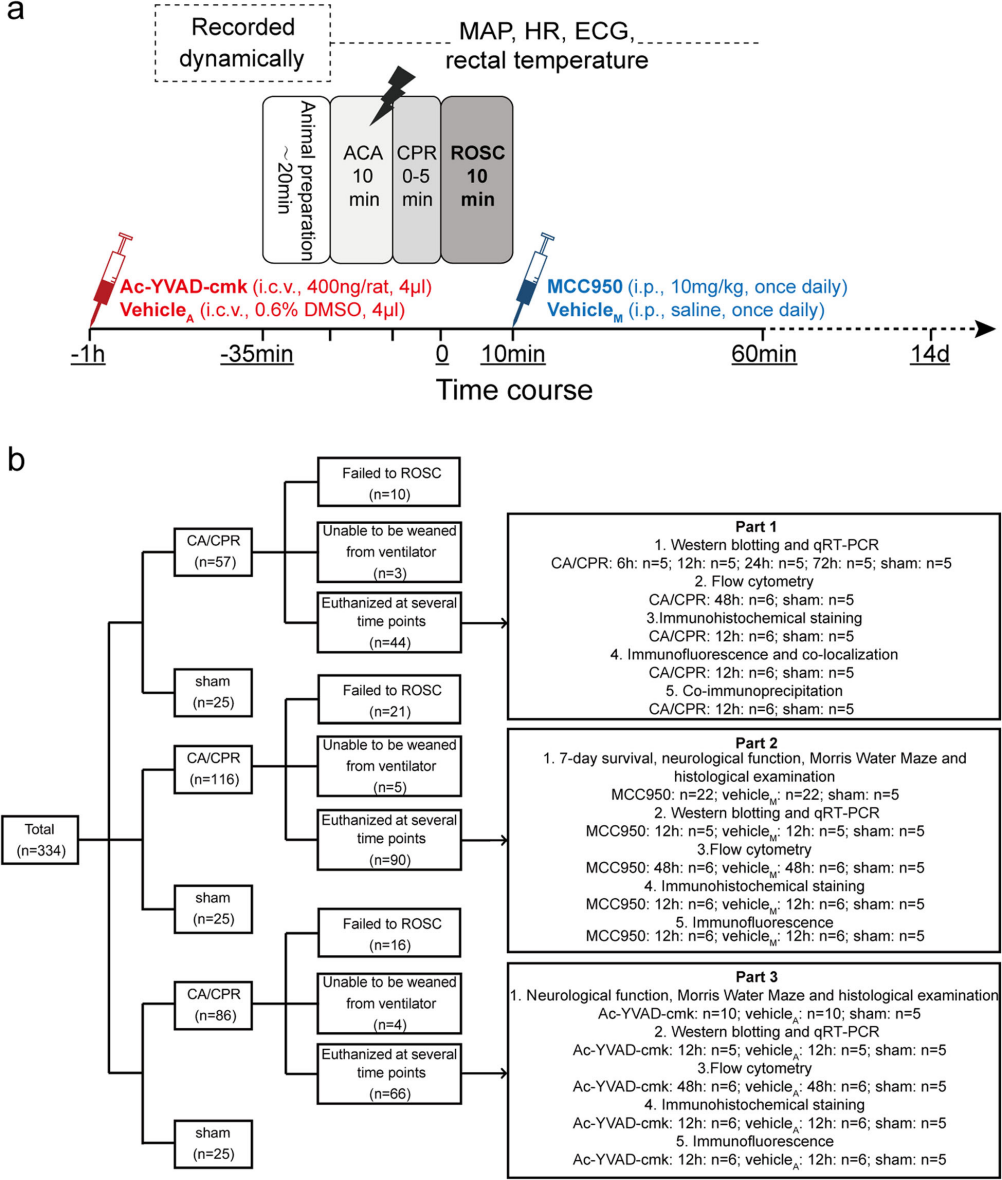
Experimental procedures and flow diagram of the study. **a** Experimental procedures, drug delivery, and measurements during baseline, asphyxial cardiac arrest, and cardiopulmonary resuscitation, and ROSC. **b** Flow diagram of the experiment. The whole study is consisted of 3 parts. *Vehicle*_*A*_ vehicle of Ac-YVAD-cmk, *Vehicle*_*M*_ vehicle of MCC950, *ECG* electrocardiogram, *HR* heart rate, *CA*/*CPR* cardiac arrest and cardiopulmonary resuscitation, *ACA* asphyxial cardiac arrest

Rats were randomly assigned to appropriate groups for different experimental purposes by using random number tables (Fig. 1). MCC950 (MedChem Express, Monmouth, NJ, USA) was dissolved in sterile saline at a concentration of 2 mg/mL (4.94 mM), and 10 mg/kg (24.72 μmol/kg) was administered intraperitoneally at 10 min after ROSC [16, 17], while rats in the vehicle group received the equivalent volume of saline. For rats utilized to assess 7-day survival rate and neurologic outcome, MCC950 was given once daily for 6 days [18]. Ac-YVAD-cmk (Sigma-Aldrich, St. Louis, MO) was dissolved in dimethyl sulfoxide (DMSO) and diluted in saline to a concentration of 100ng/μL (0.18 mM containing 0.6% DMSO), and was intracerebroventricularly (i.c.v.) injected into the right lateral ventricle of fixed rats with a loading dose of 400 ng/rat at 25–30 min before the surgeries [19], whereas rats in the vehicle group received equal volume of DMSO and saline only. Rats that underwent all procedures except asphyxial cardiac arrest and cardiopulmonary resuscitation were used as sham control.

The present study included 3 parts (Fig. 1b). In part 1, the occurrence of pyroptosis in cardiac arrest model was explored using flow cytometry, Western blotting, quantitative real-time polymerase chain reaction (qRT-PCR), co-immunoprecipitation, and histological evaluation, in which 44 rats that successfully resuscitated were implicated. In part 2, 44 rats with successful resuscitation (*n* = 22 each for MCC950 and vehicle groups) were followed up to assess survival rate, neurologic outcome, short-term memory and learning ability, and histological impairment. The other 46 rats achieving ROSC (*n* = 5 or 6 for each group) were used to illustrate the protective effect of MCC950 in the acute phase (12 h) by detecting pyroptosis and associated molecular expression. In part 3, rats were randomly allocated to receive either Ac-YVAD-cmk or an equal volume of DMSO and saline at about 30 min before surgery, and the follow-up experiments were similar to that of part 2.

### Flow cytometry

The procedure of flow cytometry was established as described previously with some adjustments [13]. Briefly, at 48-h post-surgery, all rats were anesthetized with overdose isoflurane and then perfused transcardially with ice-cold phosphate-buffered saline (PBS). The bilateral cerebral cortex and hippocampus were mechanically isolated into a single-cell suspension by passage through a 40-μm cell strainer (Falcon, Madison, WI) on ice and placed in ice-cold PBS. Afterward, cells were labeled with a FAM-FLICA assay (Immunochemistry Technologies, Bloomington, MN) to detect the caspase-1 activity, in which the FLICA reagent known as FAM-YVAD-FMK forms an irreversible covalent bond with the fluoromethyl ketone (FMK) to the cleaved caspase-1. The carboxyfluorescein (FAM) optimally excites at 488–492 nm and has a peak emission at 515–535 nm. Cell viability was assessed via the LIVE/DEAD Fixable Near-IR Dead Cell Stain (L10119, 1 μL/mL, Life Technologies) following the manufacturer’s instructions, which is featured as membrane-impermeable and combines with amine residues of membrane proteins when the cell membrane is integrated leading to a low level of fluorescence. The LIVE/DEAD Fixable Near-IR Dead Cell Stain is allowed to enter the dying cells with increased membrane permeability, thereby causing the enhancement of fluorescence in cells. After a non-specific block with CD16/CD32 antibody, the cells were further followed with surface markers CD45 Alexa 647 antibody (202212, 1.25 μg/mL, BioLegend) and CD11b v450 antibody (562108, 0.2 mg/mL, BD Horizon). Gates were established using antibody isotype controls (provided by manufacturers) and fluorescence minus one controls. The samples were acquired on LSRII/Fortessa flow cytometer (BD Biosciences, Heidelberg, Germany). Finally, the resulting flow cytometry files were analyzed with Flowjo V10 software.

### Western blotting

Western blotting was routinely performed as previously reported [20]. Mouse anti-β-actin (1:10,000, Proteintech, Chicago, IL), rabbit anti-NLRP3 (1:500, Novus Biologicals, CO, USA), rabbit anti-caspase-1 (1:500, Proteintech), mouse anti-ASC (1:500, Santa Cruz, CA, USA), rabbit anti-GSDMD (1:1000, CST, Danvers, USA), mouse anti-pro-IL-1β (1:1000, Proteintech), rabbit anti-cleaved IL-1β (1:1000, Novus Biologicals), rabbit anti-pro-IL-18 (1:1000, Proteintech), rabbit anti-cleaved IL-18 (1:300, Bioss, Beijing, China), and rabbit anti-caspase-11 (1:1000, Novus Biologicals) were used. The densities of protein blots were quantified by using ImageJ software (NIH, Bethesda, MD) and normalized to the level of β-actin.

### Measurement of gene expression

The mRNA levels of NLRP3, ASC, caspase-1, GSDMD, IL-1β, IL-18, caspase-11, and GADPH were routinely measured by qRT-PCR [20]. Relative changes of mRNA expressions were normalized to levels of GADPH.

### Survival study and neurologic function evaluation

Rats subjected to cardiac arrest modeling were followed up to 7 days, and survival rate was recorded daily. A previously validated scale of neurologic deficit score (NDS) ranging from 0 to 80 was utilized to assess the neurologic outcome at 24, 48, 72 h and 7 days after ROSC, which was performed by two investigators blinded to animal grouping [21]. The total NDS scale consisted of 7 components: general behavioral deficit, brain-stem function, motor assessment, sensory assessment, motor behavior, behavior, and seizures, in which 80 was considered normal, whereas 0 represented brain death.

### Behavioral testing

The Morris water maze was carried out to evaluate short-term spatial learning and memory as described previously [22]. The water maze apparatus is a circular tank filled with 22–24 °C opaque water rendered by black food pigment to a depth of 25 cm (divided into four quadrants, called Q1, Q2, Q3, Q4) and containing a hidden platform which is submerged 2–3 cm below the water surface and not visible to the rats at the fixed location of Q3. Firstly, the rats were trained to search for the platform on post-surgery day 9–12 at a frequency of four trials/day, orienting by referencing 3 external cues surrounding the tank. If the rats did not find the platform in 60 s, they were manually placed on it for 30 s. Rats’ movements were tracked by TSE VideoMot2 video tracking system (TSE Systems GmbH, Bad Homburg, Germany) to record the path and time taken to escape from 4 randomly assigned locations. The latency time required to locate the hidden platform was compared among groups. After the hidden platform training, the platform was removed in the probe test, and the rats had 60 s to search for the pool. The percentage of the total time that rats spent in the target quadrant and the number of platform location crossings were analyzed.

### Histological examination

Rats were euthanized after the Morris water maze experiment or predefined points (Fig. 1b), and 4-μm-thick coronal brain sections located at 3.5 mm posterior to bregma were obtained (Leica CM1800; Leica Microsystems GmbH, Heidelberg, Germany). For detecting neuronal loss, brain sections were stained with cresyl violet (Beyotime, Shanghai, China) and observed under microscope (Olympus, Tokyo, Japan). Viable neurons in the hippocampal CA1 region were those characterized as visible nucleus and intact cytoplasm with discernable and rich Nissl staining, while those with shrunken cell bodies surrounded by empty space were regarded as dead neurons.

Immunohistochemistry was conducted by incubation of sections with antibodies against neuronal nuclei (NeuN; CST) for detection of neuron, microtubule-associated protein 2 (MAP2; Sigma-Aldrich) for dendrite, glial fibrillary acidic protein (GFAP; Abcam, Cambridge, UK) for astrocyte, ionized calcium-binding adapter molecule-1 (Iba-1; Wako, Osaka, Japan) for all microglia, and CD68 (Abcam) for activated microglia. In each brain section, 3 slide-fields were randomly examined. The relative intensity area of MAP-2 immunoreactivity in hippocampus and cortex, and the number of cells with immunoreactivity of other above markers were quantified by an observer blinded to experimental grouping using Image J software (NIH).

For the immunofluorescence method, brain sections were incubated with the following primary antibodies: goat anti-NLRP3 (Abcam), mouse anti-ASC (Santa Cruz), mouse anti-caspase-1 (Santa Cruz), rabbit anti-NLRP3 (Novus), rabbit anti-caspase-1 p20 (Abcam), rabbit anti-Iba-1 (Abcam) for microglia, rabbit anti-GFAP (Abcam) for astrocytes, rabbit anti-NeuN (CST) for Neurons, rabbit anti-CD31 (Abcam) for endothelial cells, rabbit anti-Neuron Glial Antigen 2 (NG2; Proteintech) for oligodendrocyte precursor cells, and rabbit anti-oligodendrocyte lineage transcription factor 2 (Olig2; Abcam) for oligodendrocytes. The sections were then washed and detected with appropriate Alexa Fluor dye of secondary antibodies followed by counterstained with DAPI in the dark. Afterward, immunofluorescent signaling was observed with a confocal microscope (FluoView; Olympus). Digital images were recorded and analyzed using ImageJ (NIH).

### Co-immunoprecipitation

This test was performed according to the manual for Protein A/G Magnetic Beads (Bimake, Houston, USA). In brief, cerebral cortex and hippocampus were lysed in lysis buffer containing phenylmethylsulfonyl fluoride (PMSF) for IP (Genstar). Thereafter, cytoplasmic protein extraction was performed according to the manufacturer’s instructions (Beyotime). After centrifuged for 15 min at 14,000 g, 4 °C, the supernatant was obtained and quantified, of which the portion containing 500 μg protein was immunoprecipitated with 1 μg rabbit anti-NLRP3 (Abcam) or mouse anti-ASC (Santa Cruz) under rotation overnight at 4 °C. After that, immune complexes were collected, then it was incubated with the added 30 μL protein A/G magnetic beads under rotation for another 30 min at room temperature and separated magnetically. And then the beads were washed 5 times with a pH 7.5 wash buffer containing 50 mM Tris, 150 mM NaCl, 0.1% NP40, resuspended in 40 μL 1× loading buffer, and heated at 100 °C for 5 min. At the end, the protein complexes were subjected to Western blotting as described above, and the employed antibodies were as follows: rabbit anti-NLRP3 (Novus Biologicals), mouse anti-ASC (Santa Cruz), mouse anti-caspase-1 (Santa Cruz), and rabbit anti-caspase-1 (Proteintech). Whole tissue lysate prepared for IP was used as an input and homophytic IgG as the negative control. ImageJ was employed to calculate the protein ratio of NLRP3-associated ASC and pro-caspase-1 or ASC-associated NLRP3 and pro-caspase-1.

### Statistical analysis

All data were presented as means ± SD or medians and 25th to 75th percentiles (NDSs). Continuous data were analyzed with two-sided Student’s *t* test, one-way ANOVA followed by Tukey’s post hoc multiple comparison tests, or as indicated. The difference in survival rate was measured by Kaplan–Meier analysis with log-rank test. NDSs were compared with Mann–Whitney *U* test. Physiological variables and the data of escape latency in the water maze training were analyzed with repeated-measures ANOVA comprising treatment groups, time points, and treatment × time points interaction, followed by Tukey’s post hoc multiple comparison tests. SPSS 20.0 (IBM, Armonk, NY) and GraphPad Prism 6.0 (GraphPad, La Jolla, CA) were used for statistical analyses. *P* < 0.05 was considered statistically significant.

## Results

### Cardiac arrest leads to microglia activation and leukocytes infiltration in the brain

**Fig. 2:**
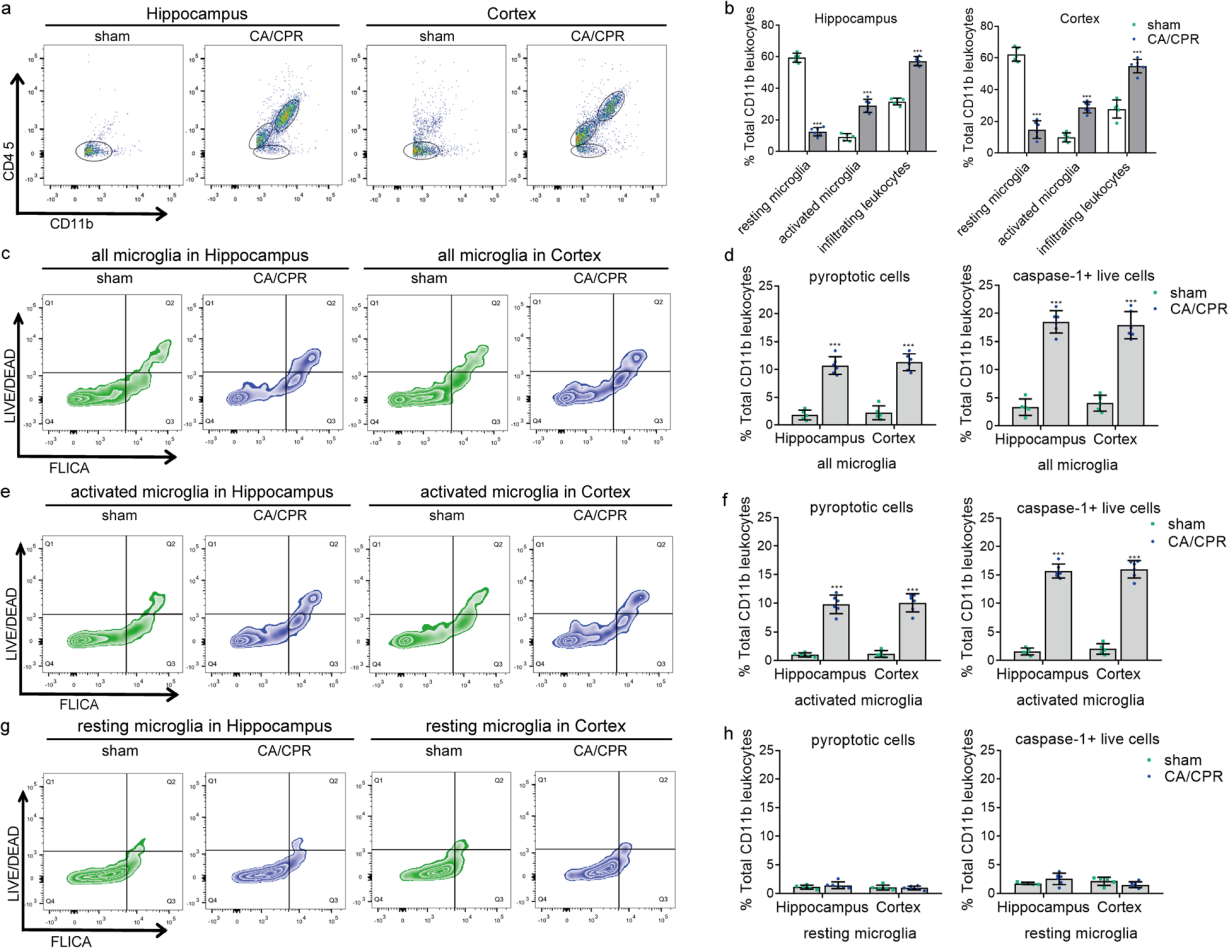
Cardiac arrest induces microglial activation, microglial pyroptosis and leukocytes infiltration. **a**, **b** Representative flow cytometry plots of all CD11b^+^ cells from sham-operated (*n* = 5) and post-cardiac arrest (*n* = 6) brain tissues of the hippocampus and cortex, accompanied by the quantification of the percentages of resting microglia, activated microglia and infiltrating CD11b+ leukocytes. **c**, **d** Representative flow cytometry plots and quantification of pyroptotic cells (Q2) and caspase-1^+^ live cells (Q3) in all microglia, labeled with LIVE/DEAD Fixable Near-IR Dead Cell Stain fluorescent probe and FAM-FLICA fluorescent probe. **e**–**h** Representative flow cytometry plots and quantification of pyroptotic cells and caspase-1^+^ live cells in the activated and resting microglia population. All data in this figure are analyzed using parametric test. Data are presented as means ± SD. ***P < 0.001 versus sham group (two-sided Student’s *t* test). *CA*/*CPR* cardiac arrest and cardiopulmonary resuscitation

Rats undergoing10-min asphyxial cardiac arrest and cardiopulmonary resuscitation modeling (*n* = 259) or sham operation (*n* = 75) were randomly assigned to appropriate groups for different experimental purposes (Fig. 1). We first sought to examine the influence of cardiac arrest on the status of microglia. At 48 h post-surgery, single-cell suspension dissociated from cortical and hippocampal brain tissues were prepared to differentiate the populations of resident microglia and infiltrating leukocytes by flow cytometry labeling with CD45 and CD11b [23, 24]. As reported [25, 26], activated microglia possess a higher level of CD45 than resting microglia, while infiltrating leukocytes own the highest amounts of CD45. Consequently, CD11b-positive cells including infiltrating myeloid-lineage leukocytes and microglia could be classified as 3 populations: low CD45 population (CD45_low_, CD11b^+^), intermediate CD45 population (CD45_int_, CD11b^+^), and high CD45 population (CD45_high_, CD11b^+^), and they may represent resting microglia, activated microglia, and invading leukocytes, respectively (Fig. 2a, b). Cells in the sham-operated hippocampus expressed a low level of CD45 (CD45_low_, CD11b^+^), manifesting as tight cell clusters on the graph with very few cells in the CD45_int_ and CD45_high_ range. As a contrast, cells in the post-cardiac arrest hippocampus revealed much higher expression of both CD45 and CD11b, reflected as looser cell clusters on the graph with many more cells in the CD45_int_ and CD45_high_ range. Similar results were observed in cells from cortical brain tissues. It indicates a large number of microglia shifts from an immuno-surveilling state to an activated state after cardiac arrest, accompanied by massive exudation of myeloid-lineage leukocytes in the post-cardiac arrest brain tissues.

### Cardiac arrest triggers microglial pyroptosis and an increase of caspase-1 activity in the activated microglia

We next investigated whether the activated microglia were correlated with pyroptosis and promoted the inflammatory response after cardiac arrest. Flow cytometry was conducted to measure the cell viability and caspase-1 activity in microglia, which were labeled with LIVE/DEAD Fixable Near-IR Dead Cell Stain fluorescent probe and FAM-FLICA fluorescent probe, respectively. The CD11b^+^ cells were at first gated into CD45_low_ CD11b^+^, CD45_int_ CD11b^+^ and CD45_high_ CD11b^+^ by CD45 labeling, as described above. Then, all microglia including resting (CD45_low_, CD11b^+^) and activated microglia (CD45_int_, CD11b^+^) were plotted using the two kinds of fluorescent probes to establish a four-quadrant gate (Fig. 2c, d): Q1, necrotic microglia (FLICA_low_, LIVE/DEAD_high_); Q2, pyroptotic microglia (FLICA_high_, LIVE/DEAD_high_); Q3, live microglia expressing caspase-1 (FLICA_high_, LIVE/DEAD_low_); Q4, live microglia without expression of caspase-1 (FLICA_low_, LIVE/DEAD_low_ ) [13].

As illustrated, the number of pyroptotic microglia and live microglia expressing caspase-1 in the post-cardiac arrest brain tissues (hippocampus and cortex) was significantly higher than that in the sham-operated brain tissues (Fig. 2c, d). Further analysis revealed that the number of cells undergoing pyroptosis and live cells expressing caspase-1 was predominantly increased in the CD45_int_ CD11b^+^ population (Fig. 2e, f) but not in the CD45_low_ CD11b^+^ population (Fig. 2g, h), indicating that the activated microglia population was the primary source of pyroptotic microglia and cells with elevated caspase-1 activity after cardiac arrest. These results imply that cardiac arrest triggers microglial pyroptosis and an increase of caspase-1 activity in the activated microglia, and the latter may also subsequently undergo pyroptosis.

### Cardiac arrest induces NLRP3 inflammasome activation in microglia

**Fig. 3:**
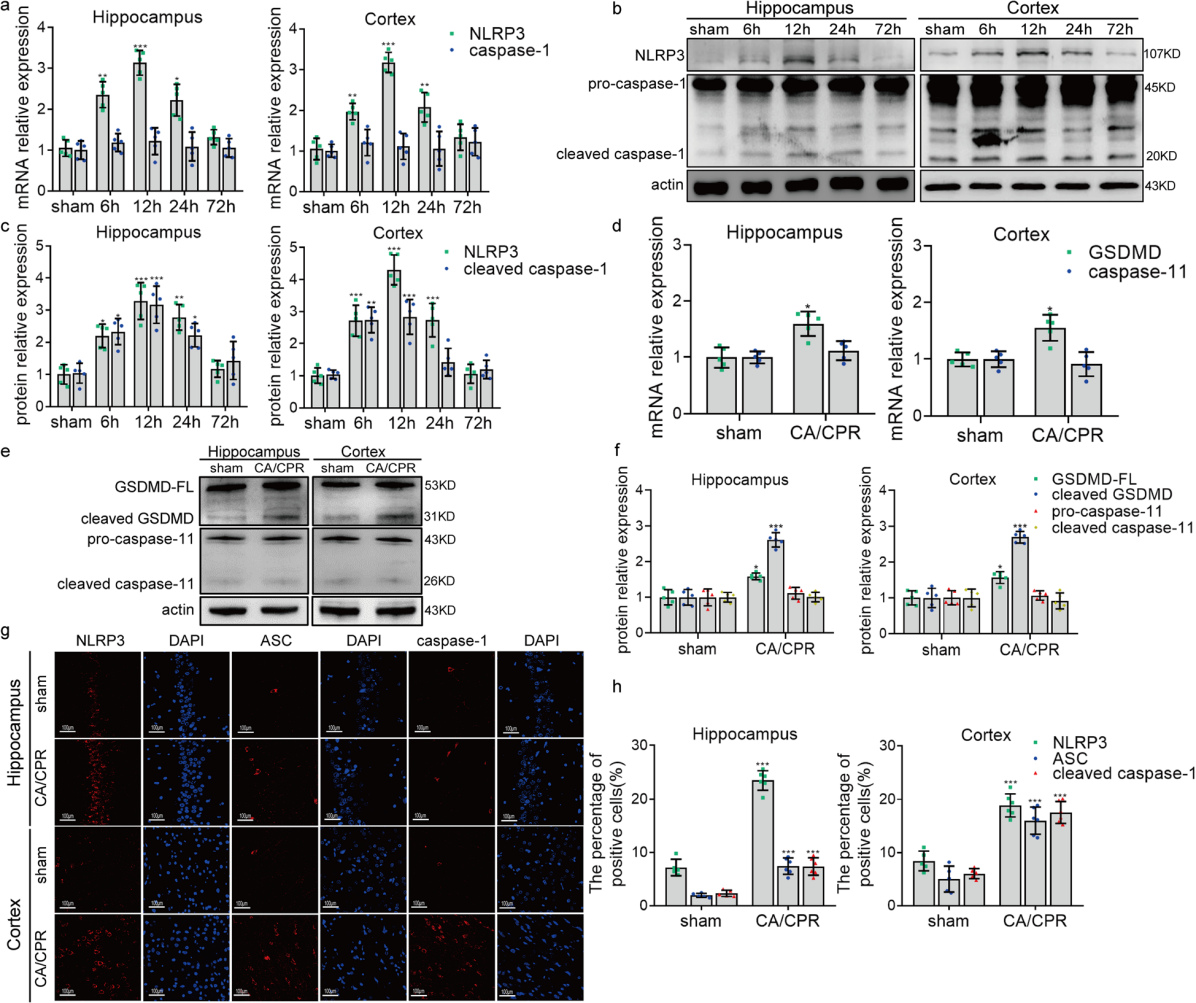
Cardiac arrest triggers the elevated level of molecules closely related to pyroptosis. **a** qRT-PCR results of the mRNA levels of NLRP3 and caspase-1 in sham-operated and post-cardiac arrest rats that were euthanized at 6, 12, 24, and 72 h after ROSC. **b**, **c** Western blotting results of NLRP3, pro-caspase-1, and cleaved caspase-1 in sham-operated and post-cardiac arrest rats that were euthanized at different time points after ROSC. **d**qRT-PCR results of the mRNA levels of GSDMD and caspase-11 expressed in hippocampus and cortex between the sham and post-surgery rats euthanized at 12 h after ROSC. **e**, **f** Western blotting results of GSDMD, cleaved GSDMD, pro-caspase-11, and cleaved caspase-11 in sham-operated and post-cardiac arrest rats. GSDMD-FL, full-length GSDMD. **g**, **h** Immunofluorescence results of NLRP3, ASC, and cleaved caspase-1 between the sham and cardiac arrest /cardiopulmonary resuscitation group, scale bar = 100 μm. All data in this figure are analyzed using parametric test. Data are represented as mean ± SD. *P < 0.05, **P < 0.01, ***P < 0.001 versus sham group. *n* = 5–6 per group. *CA*/*CPR* cardiac arrest and cardiopulmonary resuscitation

Inflammasome assembling with NLRP3 and caspase-1 is considered to be the major signaling molecule that promotes the pyroptosis in microglia [27]. Therefore, we tested the activation of NLRP3 inflammasome after cardiac arrest, aiming to provide potential targets for mediating microglial pyroptosis. Results from qRT-PCR showed that the mRNA level of NLRP3 was significantly upregulated after cardiac arrest, with a peak at the 12th hour (Fig. 3a). Consistently, the protein level of NLRP3 was elevated at 6 to 24 h after cardiac arrest (Fig. 3b, c). The mRNA level of caspase-1 and the protein level of pro-caspase-1 were not altered for the rats undergoing cardiac arrest compared to sham operation (Fig. 3a–c). However, the protein level of cleaved caspase-1, namely the active form of capase-1, was increased at 6 to 24 h in the post-cardiac arrest brain (Fig. 3b, c), indicating that the pro-caspase-1 underwent autocatalysis and activation after the cardiac arrest. In addition, the mRNA and protein levels of GSDMD were further evaluated via qRT-PCR and Western blotting to prove that the cleavage of caspase-1 here was involved in pyroptosis but not apoptosis [28]. As expected, it was observed that the protein level of cleaved GSDMD was markedly increased in the post-cardiac arrest brain, with the elevated mRNA and protein levels of GSDMD (Fig. 3d-f), which implies that caspase-1 is cleaved and activated after cardiac arrest, leading to the downstream cleavage of GSDMD and pyroptosis. We also assessed the level of caspase-11, in order to exclude the effect of caspase-11-mediated non-canonical inflammasome activation on the cleavage of GSDMD and pyroptosis [29]. It was found that there was no significant change in the protein levels of precursors of caspase-11 (pro-caspase-11) and cleaved caspase-11 of the rats in the cardiac arrest group compared to that in the sham group (Fig. 3d-f), indicating that it is the canonical NLRP3 inflammasome that mediates the microglia pyroptosis in the rats after cardiac arrest, and caspase-1 but not caspase-11 is involved in the process. The elevated expression of NLRP3 and cleaved caspase-1 were further confirmed by immunofluorescent staining at 12 h after cardiac arrest, along with ASC, another key molecule involved in the assembly of inflammasome (Fig. 3g, h).

**Fig. 4:**
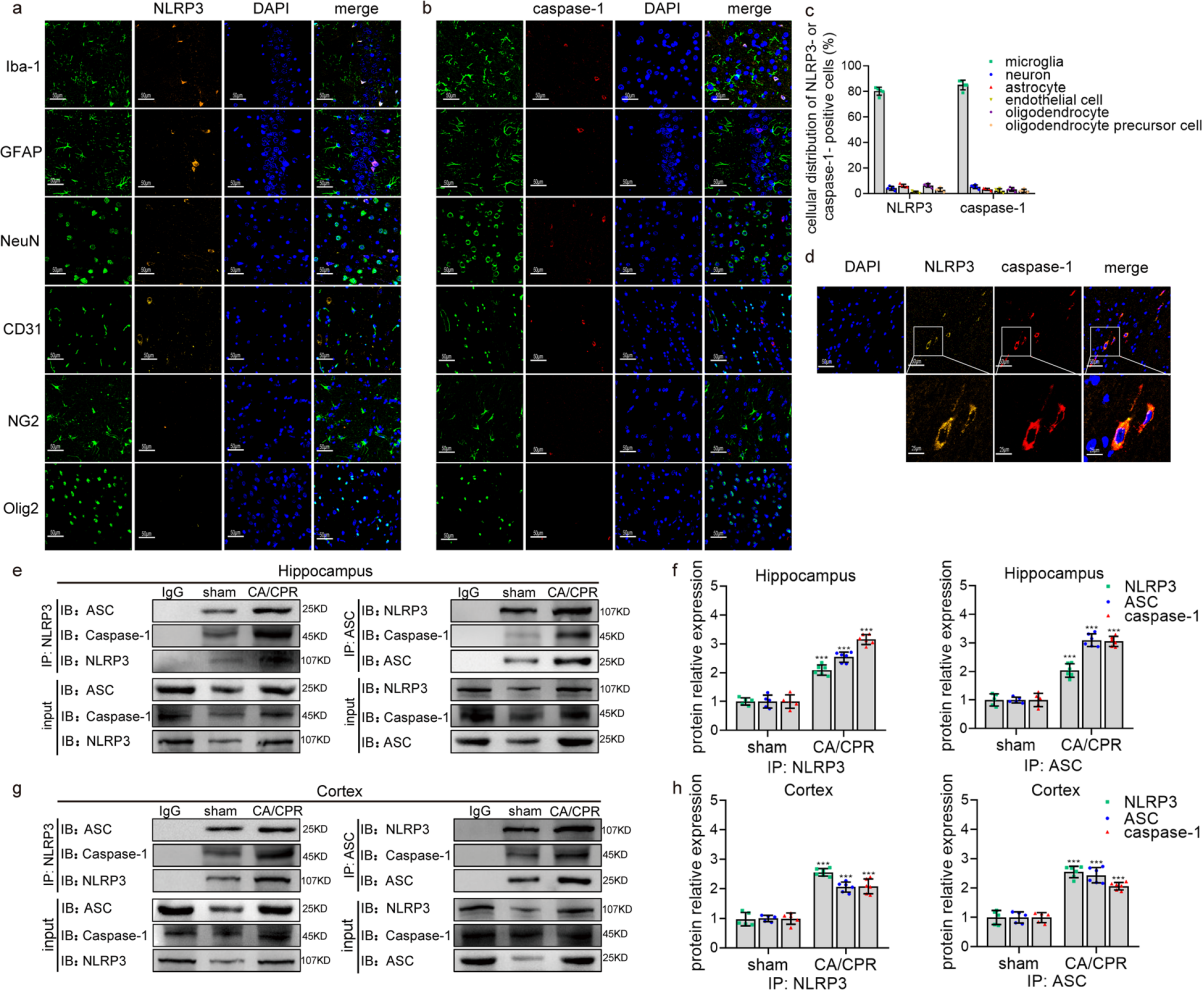
Cardiac arrest induces the activation of NLRP3 inflammasome in microglia. **a** Confocal analysis of NLRP3 (orange) and several cell markers (green) including Iba-1, GFAP, NeuN, CD31, NG2, and Olig2 staining and merged images 12 h after ROSC. **b** Confocal microscopy of caspase-1 (red) and several cell markers (green) including Iba-1, GFAP, NeuN, CD31, NG2, and Olig2 staining and merged images 12 h after ROSC. **c** The quantitative analysis of the distribution of NLRP3- or caspase-1-positive cells in above types of cells, which represents 50 cells per rat and 5 rats per group. **d** Confocal analysis of NLRP3 (orange) and caspase-1 (red) staining and merged images 12 h after ROSC. Scale bar = 50 μm or 25 μm. **e**–**h** The association of NLRP3, ASC, and caspase-1 detected by immunoprecipitation, in both cortex and hippocampus 12 h after cardiac arrest. The quantification of hippocampus (**f**) and cortex (**h**) is normalized to the level of corresponding inflammasome components from sham group. Samples from post-cardiac arrest rats are immunoprecipitated with isotype IgG as a control. All data in this figure are analyzed using parametric test. Data are represented as mean ± SD. *P < 0.05, **P < 0.01, ***P < 0.001 versus sham group. *n* = 5–6 per group. *CA*/*CPR* cardiac arrest and cardiopulmonary resuscitation

To further validate whether the assembly of inflammasome principally occurred in microglia after cardiac arrest, immunofluorescent co-localization was performed. Using confocal microscopy, we observed that NLRP3 and caspase-1 were mainly co-localized in Iba-1-positive cells but not other types of cells such as neuron, astrocytes, endothelial cells, oligodendrocyte, or oligodendrocyte precursor cells to form the inflammasome (Fig. 4a–d). It should be emphasized that since Iba-1 is not a highly specific marker, the Iba-1-positive cells, in addition to identifying microglia, may also represent a small number of invading monocytic lineage cells like macrophages. The association of NLRP3, ASC, and caspase-1 represents the intracellular activation of the inflammasome [27]. As shown by the co-immunoprecipitation (Fig. 4e-h), pairwise interactions among NLRP3, ASC, and caspase-1 were observed in both the hippocampus and cortex tissues at 12 h after cardiac arrest. Our quantitative analysis showed that the assembly of NLRP3 inflammasomes in microglia of post-surgery rats was significantly increased, featuring in the increment in the absolute amount. Interestingly, we found that there was a small amount of activated inflammasomes in sham group, which could be attributed to the effect of isoflurane anesthesia [30]. Therefore, we speculate that the combination of isoflurane and cardiac arrest in our study may lead to additional inflammasome activation over that seen in cardiac arrest without anesthesia. Taken together, these findings imply that cardiac arrest activates the assembly of NLRP3 inflammasome in microglia, which leads to the self-cleavage of caspase-1 and triggers microglial pyroptosis.

### Targeting NLRP3 with MCC950 prevents microglial pyroptosis and consequential neuroinflammation after cardiac arrest

To further test the role of NLRP3 inflammasome and correlated microglia pyroptosis in post-cardiac arrest brain injury, we targeted the NLRP3 with a highly selective inhibitor MCC950 [16]. At 10 min after ROSC from cardiac arrest, the rats were randomized to receive daily once MCC950 or vehicle until euthanasia (Fig. 1). The body weight, the time from asphyxia to cardiac arrest, the time required for ROSC, and epinephrine usage were all comparable between the MCC950 and vehicle groups (Table 1). There were also no statistical differences in physiological variables, including MAP, heart rate, and rectal temperature at baseline or after ROSC between these two groups (Table 1).

**Table 1:** Parameters of the Vehicle_M_ and MCC950 groups at baseline and during post-cardiac arrest care after ROSC

Parameters	Time points	Vehicle_M_	MCC950
Body weight, g	Baseline	377 ± 18	384 ± 20
Time from asphyxia to cardiac arrest, s	Baseline	214.0 ± 21	210.9 ± 17
Time required for ROSC, s	Baseline	90.77 ± 24	93.78 ± 27
Total dose of epinephrine, μg	Baseline	13.2 ± 3.1	12.0 ± 3.4
Mean arterial pressure, mmHg	Baseline	122 ± 8	120 ± 11
	10 min	113 ± 7	110 ± 9
	30 min	88 ± 10	91 ± 7
	60 min	85 ± 7	81 ± 9
Heart rate, beats/min	Baseline	400 ± 11	399 ± 9
	10 min	419 ± 17	424 ± 20
	30 min	364 ± 17	370 ± 16
	60 min	375 ± 14	380 ± 9
Rectal temperature, °C	Baseline	37.1 ± 0.3	37.0 ± 0.2
	10 min	37.1 ± 0.2	36.8 ± 0.3
	30 min	37.0 ± 0.3	36.9 ± 0.2
	60 min	37.0 ± 0.2	36.9 ± 0.3

Physiological variables are measured at baseline and at 10, 30, and 60 min after ROSC. The values are expressed as mean ± SD. *Vehicle*_*M*_ vehicle of MCC950

**Fig. 5:**
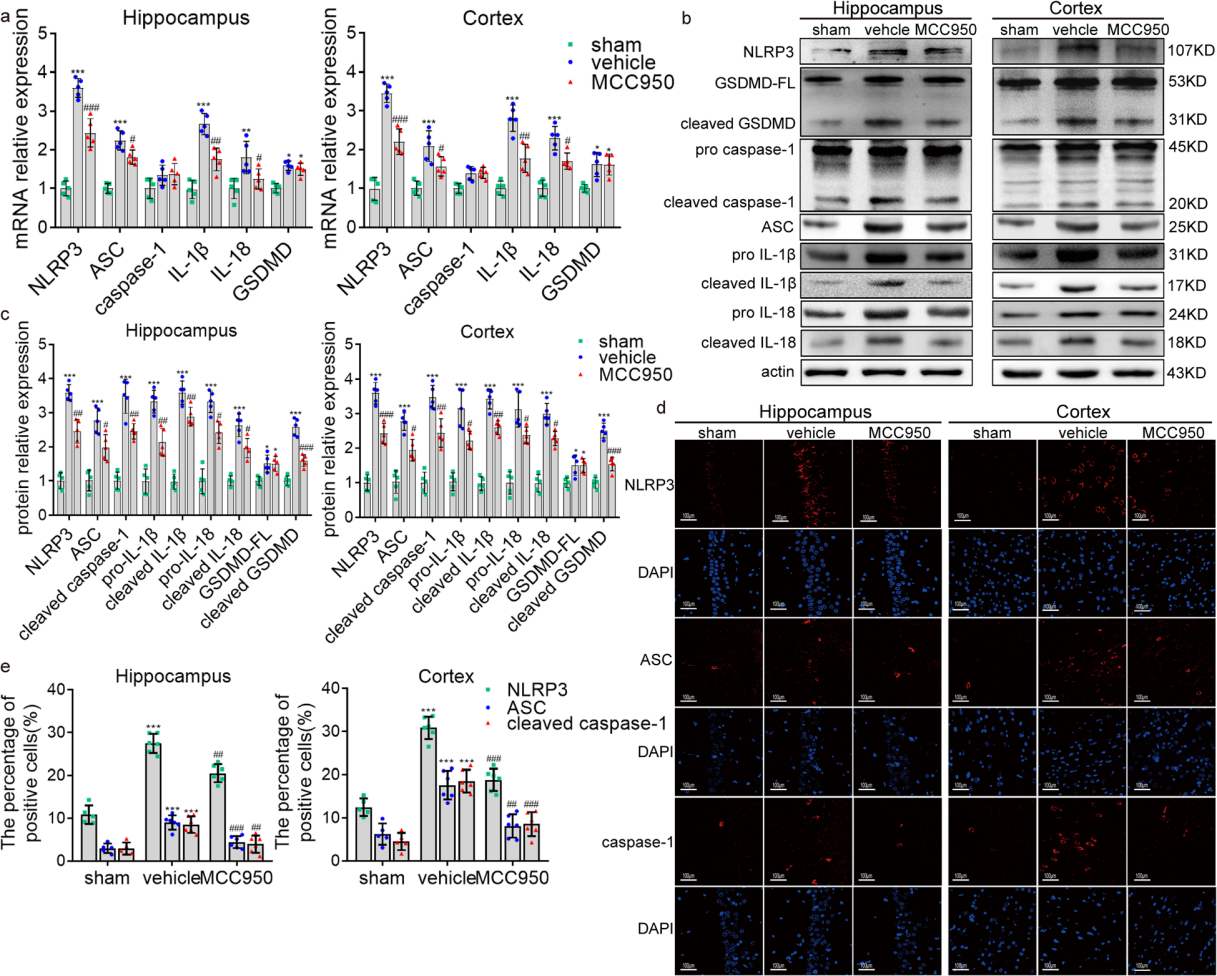
The effect of MCC950 on the level of molecules associated with pyroptosis. **a**–**c** qRT-PCR and Western blotting results showing the mRNA levels (**a**) and protein levels (**b**, **c**) of NLRP3, ASC, caspase-1, GSDMD, IL-1β, and IL-18 among the sham, vehicle, and MCC950 groups. GSDMD-FL, full-length GSDMD. **d**, **e** Immunofluorescence staining results showing the protein expression of NLRP3, ASC, and cleaved caspase-1 among the sham, vehicle, and MCC950 groups. All data in this figure are analyzed using parametric test. Data are presented as mean ± SD. Statistical significances are determined with one-way ANOVA followed by Tukey’s post hoc test. *P < 0.05, **P < 0.01, ***P < 0.001 versus sham; #*P* < 0.05, ##*P* < 0.01, ###*P* < 0.001 versus vehicle. *n* = 5–6 per group

Given the direct bioactivity of MCC950 on NLRP3, we first examined the influence of MCC950 on the assembly of NLRP3 inflammasome, the cleavage of caspase-1 and GSDMD, and the maturation of IL-1β and IL-18. The results from qRT-PCR and Western blotting showed that MCC950 significantly suppressed the levels of NLRP3, ASC, IL-1β, and IL-18 at 12 h after cardiac arrest, in both hippocampus and cortex (Fig. 5a–c). Also, MCC950 markedly inhibited the activity of cleaved caspase-1 but not the level of pro-caspase-1, suggesting that MCC950 suppresses the activation but not the generation of caspase-1. Besides, we found the similar result that MCC950 could significantly inhibit the activation of GSDMD and reverse the increased level of cleaved GSDMD after surgery, with no effect on the level of full-length GSDMD (Fig. 5a–c). Consistently, less NLRP3-positive, ASC-positive, and cleaved caspase-1-positive cells were observed in the MCC950-treated group (Fig. 5d, e).

**Fig. 6:**
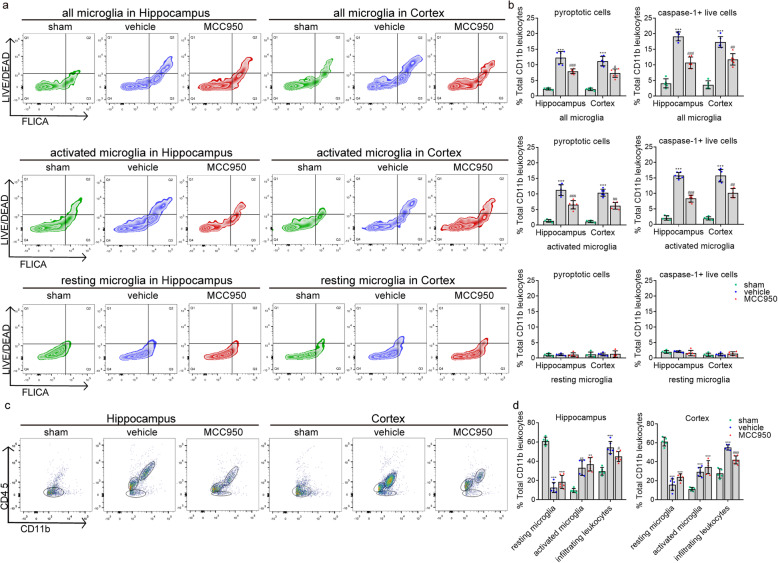
The effect of MCC950 on microglial pyroptosis and caspase-1 activity. **a**, **b** Detection of pyroptotic cells (FLICA_high_, LIVE/DEAD_high_) and caspase-1^+^ live cells (FLICA_high_, LIVE/DEAD_low_) in all microglia, activated microglia, and resting microglia population, respectively. **c**, **d** Detection of infiltrating CD11b^+^ leukocytes, resting and activated microglia from the cortex and hippocampus of sham, vehicle, and MCC950 groups at 48 h after ROSC. All data in this figure are analyzed using parametric test. Data are presented as mean ± SD. Statistical significances are determined with one-way ANOVA followed by Tukey’s post hoc test. *P < 0.05, **P < 0.01, ***P < 0.001 versus sham; #*P* < 0.05, ##*P* < 0.01, ###*P* < 0.001 versus vehicle. *n* = 5–6 per group

We then examined whether MCC950 prevents microglial pyroptosis and the increased caspase-1 activity in activated microglia caused by cardiac arrest. In consistent with the above results, a reduced number of live cells expressing caspase-1 and pyroptotic cells were observed in the MCC950 but not the vehicle groups at 48 h after cardiac arrest (Fig. 6a, b). MCC950 treatment significantly reduced the number of pyroptotic cells and live cells expressing caspase-1 compared to the vehicle group in the CD45_int_ CD11b^+^ population (Fig. 6a, b). However, there was no difference in the number of pyroptotic and caspase-1-positive cells between the MCC950-treated and the vehicle-treated groups in the CD45_low_ CD11b^+^ population (Fig. 6a, b). Moreover, the delivery of MCC950 led to a significant reduction in the number of CD45_high_ CD11b^+^ cells (Fig. 6c, d) accompanied by the inhibition of IL-1β and IL-18, suggesting that the intervention of NLRP3 by MCC950 prevents the post-cardiac arrest inflammatory response. Strikingly, MCC950 inhibited not only the mature IL-1β and IL-18 but also their precursors, which could be attributed to the overall suppression of inflammatory response and thus reduced the transcription of these two cytokines by nf-κb [31–33]. Therefore, our findings provide evidence that targeting NLRP3 by MCC950 suppresses the occurrence of microglial pyroptosis and consequential inflammatory response after cardiac arrest.

### Targeting NLRP3 with MCC950 improves survival and neurologic outcome after cardiac arrest

**Fig. 7:**
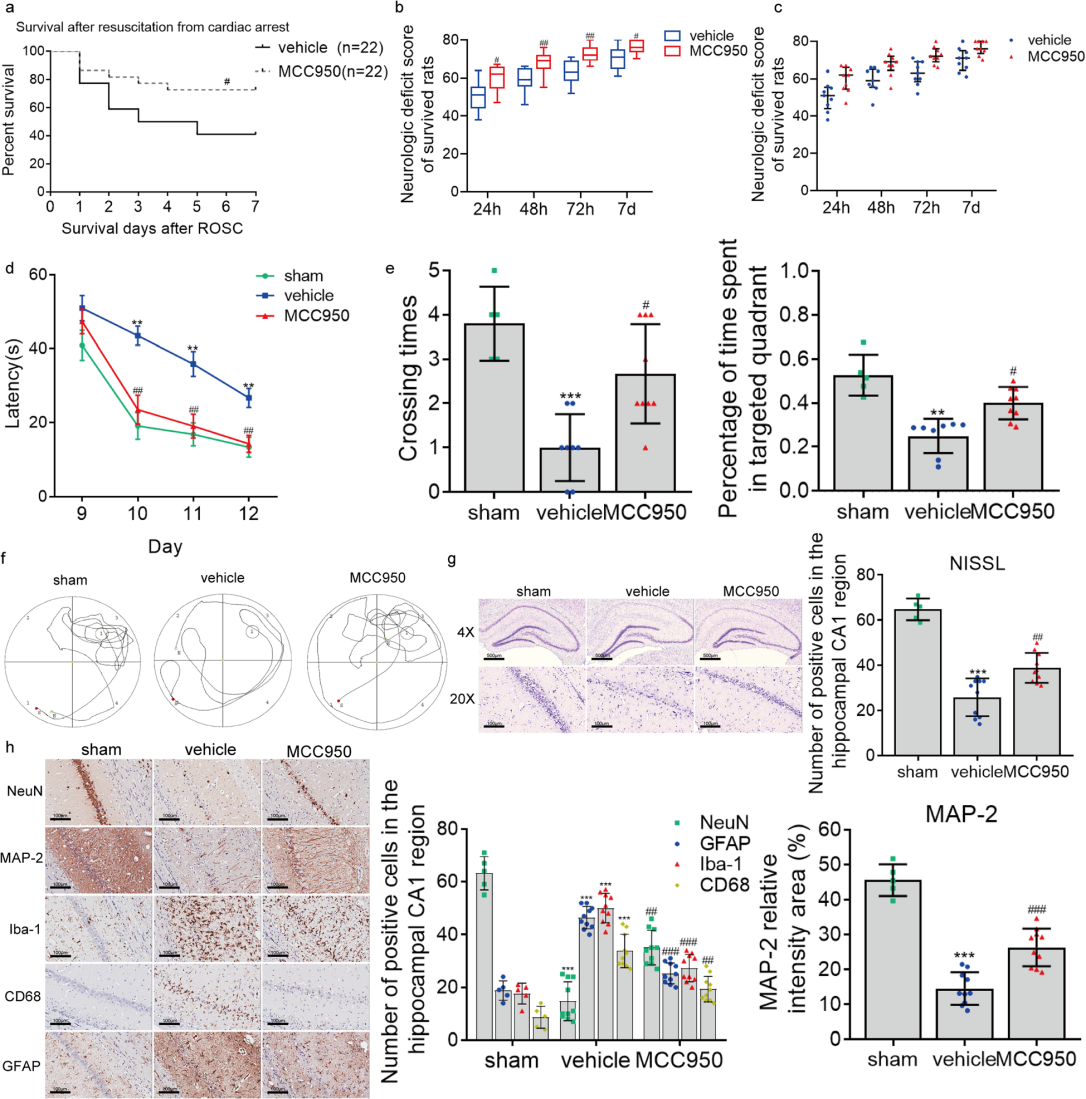
MCC950 treatment improves survival, neurologic outcome, neurocognitive function, and neuropathological damage after cardiac arrest. **a** Kaplan–Meier analyses of cumulative survival during 7-day follow-up after ROSC. Solid line, vehicle group (*n* = 22); dashed line, MCC950 group (*n* = 22). **b** Neurologic deficit scores (NDS; 0 = brain death; 80 = normal) of survived rats at 24, 48, 72 h, and on day 7 after ROSC. **c** Scatter plots reflecting the difference in distribution of NDSs between the vehicle and MCC950 groups. **d**–**f** The effect of MCC950 on post-cardiac arrest spatial memory and learning deficits assessed by Morris water maze analysis, including the mean latency in searching the hiding platform (**d**), the frequency of crossing the platform area, and percentage of time spent in the target quadrant (Q3) during the probe trial (**e**, **f**). **g**, **h** The effect of MCC950 on post-cardiac arrest neuropathological damage characterized by the changes in Nissl staining and immunohistochemical staining for NeuN, GFAP, MAP-2, Iba-1, and CD68. Scale bar = 500 μm or 100 μm. The data in panels **a**, **b**, and **c** are analyzed using non-parametric test, and the other data are analyzed via parametric test. Data are presented as mean ± SD or medians and 25th to 75th percentiles (NDSs). Statistical significances are determined with one-way ANOVA followed by Tukey’s post hoc test or by Mann–Whitney *U* test (NDSs). *P < 0.05, **P < 0.01, ***P < 0.001 versus sham (*n* = 5); #*P* < 0.05, ##*P* < 0.01, ###*P* < 0.001 versus vehicle

We then explored whether the suppression of microglia pyroptosis and consequential inflammatory response improved outcomes by targeting NLRP3 with MCC950 in cardiac arrest modeling rats. We found that the 7-day survival rate in the MCC950 group (72.7%, 16 of 22) was significantly higher than that in the vehicle group (40.9%, 9 of 22) (Fig. 7a). In addition, we used the NDS to assess the neurologic function after cardiac arrest and found that rats in the vehicle group presented lower NDSs at 24, 48, 72 h and on day 7 after ROSC than those in the MCC950 group (Fig. 7b, c), indicating that the neurologic deficit caused by cardiac arrest was alleviated by MCC950 treatment.

We also performed the Morris water maze test to evaluate the effect of MCC950 on short-term spatial learning and memory ability after cardiac arrest. As a comparison, sham-operated rats were used in this part of the experiment. During the hidden-platform training on day 9 to day 12 after surgery, rats in the sham, vehicle, and MCC950 groups all showed a gradual decline in latency to find the hidden platform over time (*F* = 60.086, *P* = 0.000) (Fig. 7d). In addition, analysis of the training data by repeated-measures ANOVA showed that escape latency differed significantly among the groups (*F* = 13.662, *P* = 0.000), with no significant interaction between groups and time points (*F* = 2.455, *P* = 0.113). Compared with sham operation, cardiac arrest led to extended latencies on day 10 to day 12 in the vehicle group (all *P* < 0.05 vs. sham), whereas the extended latencies were partly prevented by MCC950 treatment (all *P* < 0.05 vs. vehicle) using the Tukey’s post hoc test. The poor performance of vehicle animals to climb up the hidden platform should not be attributed to slower swimming speed because the mean swimming speed was comparable among the three groups (data not shown). On day 13 after cardiac arrest, all rats received the probe test to assess their short-term memory (Fig. 7e, f). Results showed that the frequency of crossing the platform area was lower in the vehicle group than the sham group, whereas this figure was increased after MCC950 treatment. Besides, rats administrated with MCC950 spent markedly more time searching in the platform quadrant (Q3) in comparison with vehicle rats, while vehicle-operated rats spent increased time in the other three non-platform quadrants (Fig. 7e, f). These findings from Morris water maze test indicate that MCC950 treatment rescued the spatial learning and memory deficiency caused by cardiac arrest.

These results demonstrate that long-term treatment with MCC950 after cardiac arrest provides persistent neuroprotection to improve 7-day survival and neurologic outcome.

### Targeting NLRP3 with MCC950 ameliorates histological injury after cardiac arrest

To assess the histological damage caused by cardiac arrest and explore the profound effect of MCC950, all the rats involved in the Morris water maze test were euthanized on day 14 after cardiac arrest to conduct Nissl and immunohistochemical staining (Fig. 7g, h). The results from Nissl staining demonstrated that cardiac arrest induced neuron loss in the hippocampal CA1 region, a vulnerable region to global ischemia, whereas the neuron loss was partially restored by MCC950 (Fig. 7g). Consistently, less NeuN^+^ cells (neuron) were noticed in the vehicle group compared to the sham group, while a significantly increased number of NeuN^+^ cells was found in the MCC950 group. We also examined the injury to dendrite by immunostaining for MAP-2, a protein that was enriched in neuronal dendrites and acted as a stabilizing molecule for the dendritic cytoskeletal integrity (Fig. 7h). Results showed that cardiac arrest caused a dramatic dendritic loss in the vehicle group, as compared with sham controls. However, the dendritic loss was markedly reversed after MCC950 treatment. These findings, therefore, indicate that MCC950 substantially prevents the neuron loss and dendritic injury induced by cardiac arrest.

Neuron loss is usually accompanied by glial activation to clean up the cell debris. As illustrated in Fig. 7h, microglia and astrocytes were dramatically activated in the post-cardiac arrest hippocampal CA1 region, as evidenced by increased immunoreactivities of Iba-1 for microglia and GFAP for astrocytes. The number of Iba-1-positive microglia and GFAP-positive astrocytes were both markedly reduced by MCC950 intervention compared to the vehicle group, indicating that the activation of microglia and astrocytes were inhibited by MCC950. Based on the results of flow cytometry, the number of activated microglia was elevated after cardiac arrest modeling, which was perceived as an important link to the pyroptosis implicated in the vigorous post-cardiac arrest inflammation and subsequent brain damage. We thus stained the brain sections with CD68 antibody, a marker for activated microglia, to further explore the inhibitory effect of MCC950 on the activated microglia. Unlike the findings observed at 48 h after cardiac arrest, there was a significant decrease in the intensity of CD68 staining in the presence of MCC950 treatment after cardiac arrest (Fig. 7h). This might be due to the inhibition of early inflammatory response by MCC950 after cardiac arrest, thereby reducing the further activation of microglia. Taken together, these results suggest that MCC950 significantly prevents histological injuries, which might be via interfering NLRP3 inflammasome activation and suppressing microglia pyroptosis in the brain after cardiac arrest.

### Targeting caspase-1 by Ac-YVAD-cmk prevents microglial pyroptosis and consequential neuroinflammation after cardiac arrest

To further verify the role of NLRP3 inflammasome in mediating post-cardiac arrest microglial pyroptosis and its consequential brain injury, we used a selective inhibitor Ac-YVAD-cmk to target caspase-1, the canonical executor of pyroptosis [34]. In this part, rats were randomized to receive one dose of Ac-YVAD-cmk (400 ng in 4 μL, i.c.v.) or vehicle before the induction of asphyxial cardiac arrest (Fig. 1). There was no significant difference found in the ratio of achieving ROSC between the Ac-YVAD-cmk (35/42, 83.3%) and vehicle (35/44, 79.5%) groups. Moreover, the body weight, the time from asphyxia to cardiac arrest, the time required for ROSC, epinephrine usage, and physiological variables were all comparable between these two groups (Table 2).

**Table 2:** Parameters of the of the Vehicle_A_ and Ac-YVAD-cmk groups at baseline and during post-cardiac arrest care after ROSC

Parameters	Time points	Vehicle_A_	Ac-YVAD-cmk
Body weight, g	Baseline	381 ± 13	374 ± 17
Time from asphyxia to cardiac arrest, s	Baseline	220.4 ± 15	217.1 ± 23
Time required for ROSC, s	Baseline	99.06 ± 17	96.45 ± 22
Total dose of epinephrine, μg	Baseline	12.3 ± 3.1	11.1 ± 3.0
Mean arterial pressure, mmHg	Baseline	119 ± 7	124 ± 11
	10 min	116 ± 12	107 ± 7
	30 min	84 ± 9	83 ± 5
	60 min	80 ± 5	81 ± 11
Heart rate, beats/min	Baseline	407 ± 11	403 ± 7
	10 min	431 ± 15	427 ± 19
	30 min	372 ± 11	369 ± 17
	60 min	383 ± 17	379 ± 15
Rectal temperature, °C	Baseline	36.8 ± 0.4	36.9 ± 0.2
	10 min	37.1 ± 0.3	36.9 ± 0.3
	30 min	37.0 ± 0.2	36.8 ± 0.3
	60 min	36.8 ± 0.2	36.9 ± 0.2

Physiological variables are measured at baseline and at 10, 30, and 60 min after ROSC. The values are expressed as mean ± SD. *Vehicle*_*A*_ vehicle of Ac-YVAD-cmk

**Fig. 8:**
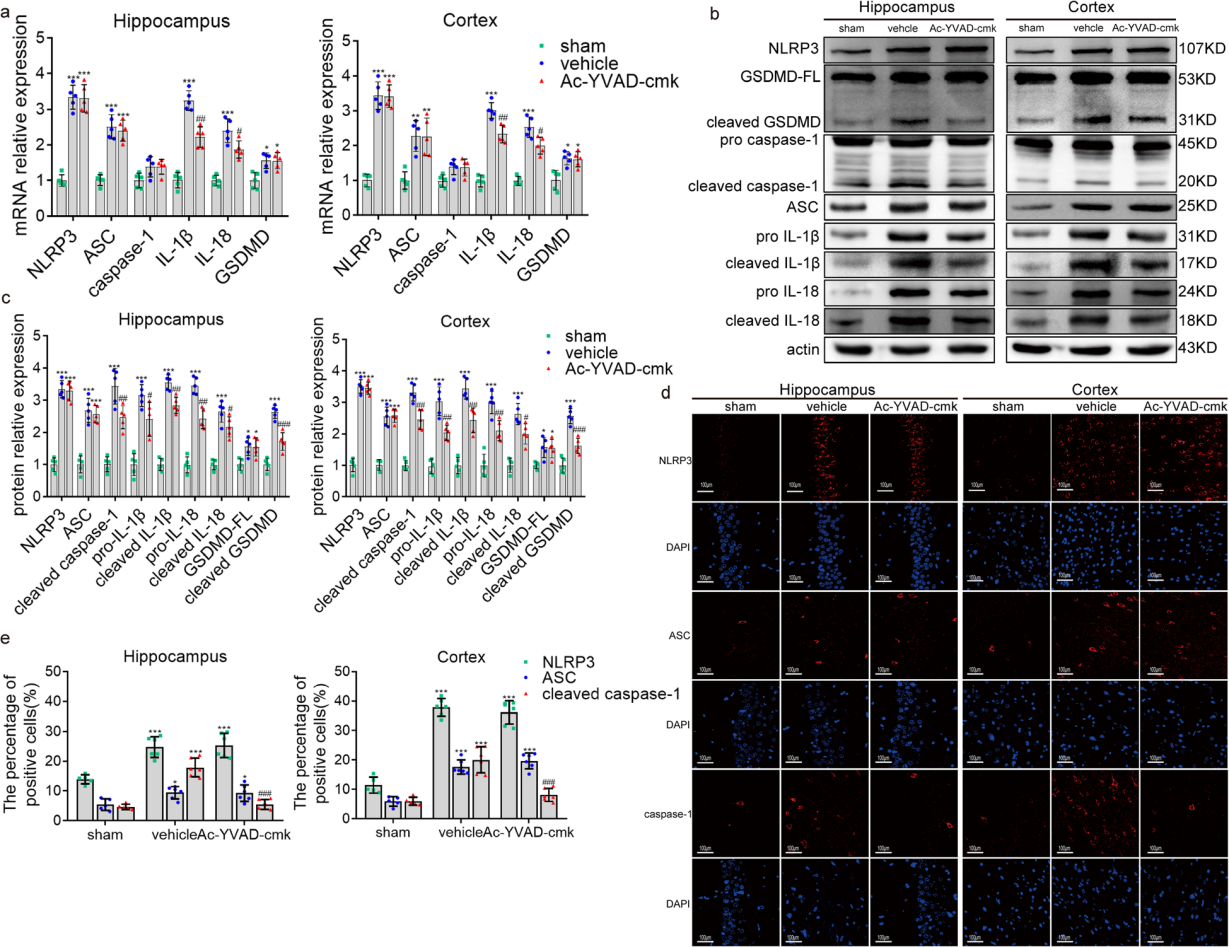
The effect of Ac-YVAD-cmk on the level of molecules associated with pyroptosis. **a**–**c** qRT-PCR and Western blotting results showing the mRNA levels (**a**) and protein levels (**b**, **c**) of NLRP3, ASC, caspase-1, GSDMD, IL-1β, and IL-18 among the sham, vehicle, and Ac-YVAD-cmk groups. GSDMD-FL, full-length GSDMD. **d**, **e** Immunofluorescence staining results showing the protein expression of NLRP3, ASC, and cleaved caspase-1 among the sham, vehicle and Ac-YVAD-cmk groups. All data in this figure are analyzed using parametric test. Data are presented as mean ± SD. Statistical significance was determined with one-way ANOVA followed by Tukey’s post hoc test. *P < 0.05, **P < 0.01, ***P < 0.001 versus sham; #*P* < 0.05, ##*P* < 0.01, ### *P* < 0.001 versus vehicle. *n* = 5–6 per group

We found that the mRNA levels coding for IL-1β and IL-18 in hippocampus and cortex of post-cardiac arrest rats were downregulated after treatment with Ac-YVAD-cmk. However, there was no significant change for the mRNA levels of NLRP3, ASC, caspase-1, and GSDMD with Ac-YVAD-cmk treatment (Fig. 8a). Ac-YVAD-cmk treatment also significantly inhibited the activity of cleaved caspase-1 and cleaved GSDMD, and decreased the protein level of pro-IL-1β, pro-IL-18, IL-1β, and IL-18 in both hippocampus and cortex, while the levels of NLRP3 and ASC were not changed after Ac-YVAD-cmk treatment (Fig. 8b, c). These results were further confirmed by immunofluorescence staining. Inhibition of caspase-1 by Ac-YVAD-cmk dramatically reduced cleaved caspase-1 but not NLRP3 or ASC, as compared with those vehicle controls (Fig. 8d, e). These findings suggest that Ac-YVAD-cmk restrains the cleavage of caspase-1, and thereby prevents the maturation of IL-1β and IL-18 in the brain after cardiac arrest.

**Fig. 9:**
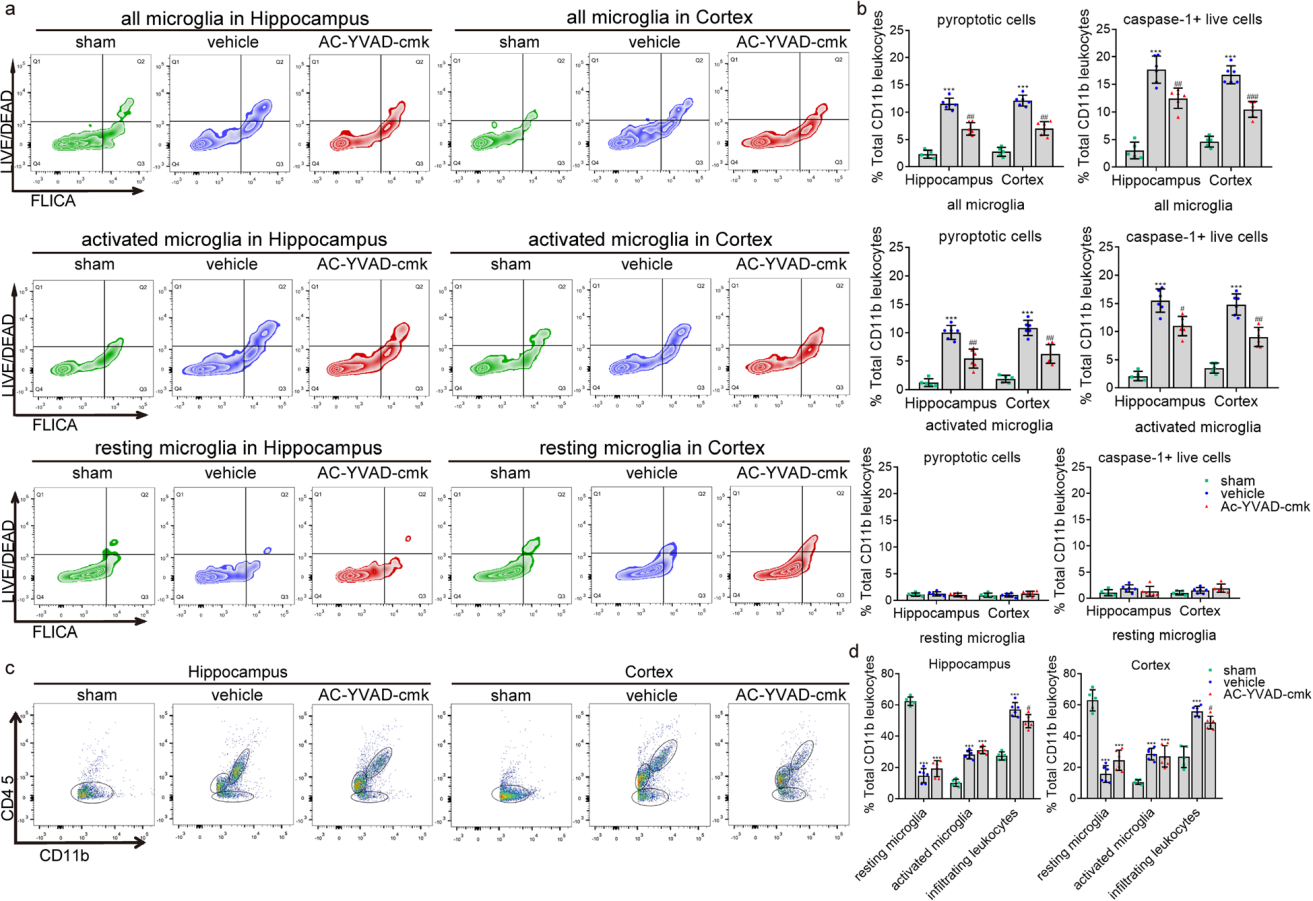
The effect of Ac-YVAD-cmk on microglial pyroptosis and caspase-1 activity. **a**, **b** Detection of pyroptotic cells (FLICA_high_, LIVE/DEAD_high_) and caspase-1^+^ live cells (FLICA_high_, LIVE/DEAD_low_) in all microglia, activated microglia, and resting microglia population, respectively. **c**, **d** Detection of infiltrating CD11b^+^ leukocytes, resting, and activated microglia from the cortex and hippocampus of sham, vehicle, and Ac-YVAD-cmk groups. All data in this figure are analyzed using parametric test. Data are presented as mean ± SD. Statistical significance was determined with one-way ANOVA followed by Tukey’s post hoc test. *P < 0.05, **P < 0.01, ***P < 0.001 versus sham; #*P* < 0.05, ##*P* < 0.01, ###*P* < 0.001 versus vehicle. *n* = 5–6 per group

We next detected whether the inhibition of caspase-1 by Ac-YVAD-cmk suppressed microglia pyroptosis and the caspase-1 activity in activated microglia caused by cardiac arrest. As expected, targeting caspase-1 by Ac-YVAD-cmk substantially reduced the number of pyroptotic cells and live cells expressing caspase-1 in all microglia population (CD45_low_ CD11b^+^ and CD45_int_ CD11b^+^) as well as in the CD45_int_ CD11b^+^ population after cardiac arrest (Fig. 9a, b). Again, the suppression of microglial pyroptosis by Ac-YVAD-cmk led to a notably decreased number of CD45_high_ CD11b^+^ population compared to the vehicle group (Fig. 9c, d). Thus, targeting caspase-1 by Ac-YVAD-cmk prevents microglial pyroptosis and consequential neuroinflammation after cardiac arrest.

### Targeting caspase-1 by Ac-YVAD-cmk ameliorates neurological injury after cardiac arrest

**Fig. 10:**
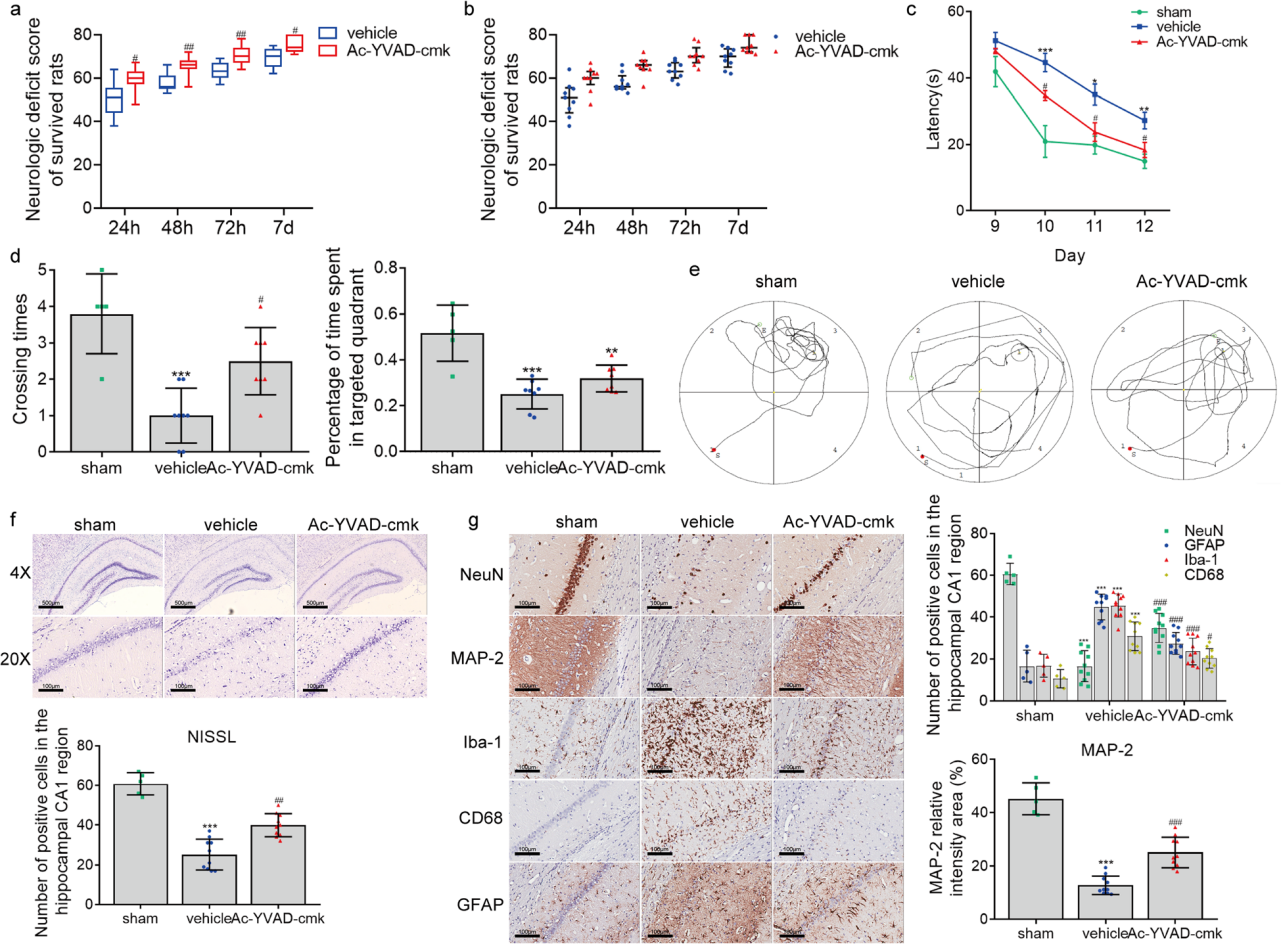
Ac-YVAD-cmk treatment improves neurologic outcome, neurocognitive function and neuropathological damage after cardiac arrest.** a** NDSs of survived rats at 24, 48, 72 h, and on day 7 after ROSC. **b** Scatter plots reflecting the difference in distribution of NDSs between the vehicle and Ac-YVAD-cmk groups. **c**–**e** The effect of Ac-YVAD-cmk on post-cardiac arrest spatial memory and learning deficits assessed by Morris water maze analysis, including the mean latency in searching the hiding platform (**c**), the frequency of crossing the platform area and percentage of time spent in the target quadrant (Q3) during the probe trial (**d**, **e**). **f**, **g** The effect of Ac-YVAD-cmk on post-cardiac arrest neuropathological damage characterized by the changes in Nissl staining and immunohistochemical staining for NeuN, GFAP, MAP-2, Iba-1, and CD68. Scale bar = 500 μm or 100 μm. The data in panel a and b are analyzed using non-parametric test, and the other data are analyzed via parametric test. Data are presented as mean ± SD or medians and 25th to 75th percentiles (NDSs). Statistical significances are determined with one-way ANOVA followed by Tukey’s post hoc test or by Mann–Whitney *U* test (NDSs). *P < 0.05, **P < 0.01, ***P < 0.001 versus sham; #*P* < 0.05, ##*P* < 0.01, ###*P* < 0.001 versus vehicle. *n* = 5–10 per group

We then assessed whether the suppression of microglia pyroptosis by targeting caspase-1 with Ac-YVAD-cmk ameliorated neurological injury in a rat model of cardiac arrest. Post-cardiac arrest rats in the Ac-YVAD-cmk group exhibited statistically higher NDSs at 24, 48, 72 h, and 7 days than those in the vehicle group (Fig. 10a, b), suggesting that Ac-YVAD-cmk intervention reduced neurologic damage. Furthermore, during the training period of the Morris water maze test, the escape latency became progressively shorter in all groups over time (*F* = 71.673, *P* = 0.000), and repeated-measures ANOVA revealed that escape latency differed significantly among the groups (*F* = 13.768, *P* = 0.000), with no significant interaction between groups and time points (*F* = 2.040, *P* = 0.075). Besides, Tukey’s post hoc test showed that Ac-YVAD-cmk substantially shorted the latency in finding the hidden platform on day 10, 11, and 12 after cardiac arrest modeling groups (Fig. 10c), while the mean swimming speed was comparable between the Ac-YVAD-cmk and the vehicle groups (data not shown). In the probe trial of the Morris water maze test, the frequency of crossing the platform area was increased in the Ac-YVAD-cmk-treated group compared with the vehicle-treated groups (Fig. 10d, e). Moreover, rats with an intracerebroventricular injection of Ac-YVAD-cmk had a trend toward spending more time searching in the platform quadrant (Q3) in comparison with vehicle rats, while vehicle-operated rats seemed to spend increased time in the other three non-platform quadrants (Fig. 10d, e).

After the Morris water maze test, all rats in this part of the experiment were euthanized, and brain sections were prepared for histological injury evaluation. Our results of Nissl staining revealed that there were significantly more viable neurons in the Ac-YVAD-cmk-treated group compared to the vehicle group (Fig. 10f). Ac-YVAD-cmk-treated rats exhibited more NeuN^+^ neurons in hippocampal CA1 region than vehicle-treated rats (Fig. 10g). Besides, post-cardiac arrest rats in the vehicle group exhibited extensive loss of MAP2-immunoreactive dendrites in the hippocampus CA1 region, which was partially recovered by Ac-YVAD-cmk treatment (Fig. 10g). We next carried out the Iba-1, CD68, and GFAP immunohistochemical staining to verify the activation of microglia and astrocytes. As expected, extensive Iba-1-positive, GFAP-positive, and CD68-positive cells appeared in the hippocampal CA1 region in the post-cardiac arrest rats treated with vehicle solution compared to the sham controls. However, Ac-YVAD-cmk treatment significantly reduced Iba-1-positive, GFAP-positive, and CD68-positive cells in the hippocampal CA1 region of post-cardiac arrest rats. These results suggest that pharmacological blockade of caspase-1 by Ac-YVAD-cmk considerably suppressed cardiac arrest-induced activation of microglia and astrocytes. Overall, the findings from this part suggest that pre-treatment with Ac-YVAD-cmk provides profound effects in preventing neurologic deficit and alleviating neuropathological injury induced by cardiac arrest, which could be attributed to the suppression of microglial pyroptosis and ensuing inflammatory responses.

## Discussion

Asphyxia caused by foreign-body airway obstruction, hanging, or strangulation is a common pathogenesis of cardiac arrest [35]. In this study, we used a 10-min asphyxial cardiac arrest and cardiopulmonary resuscitation model to reproduce the pathophysiology of hypoxic-ischemic encephalopathy and histological injury similar to asphyxial humans [36, 37]. We demonstrate that (1) cardiac arrest leads to microglial activation and leukocytes infiltration in the brain; (2) cardiac arrest triggers microglial pyroptosis and increases the caspase-1 activity in the activated microglia population, which is mediated by the NLRP3 inflammasome; and (3) targeting NLRP3 and caspase-1 with MCC950 and Ac-YVAD-cmk significantly prevent microglial pyroptosis and consequential inflammatory response after cardiac arrest, which further leads to improved neurologic outcome and less histological injury in cardiac arrest-modeling rats. Our results, for the first time, highlight the significance of NLRP3 inflammasome-mediated microglial pyroptosis in the development of post-cardiac arrest brain injury.

Sterile inflammation has been increasingly recognized as an important factor leading to secondary brain injury in a variety of neuropathic conditions, where the activation of microglia, the resident immune cells in the brain, is the initial step in the inflammatory responses [38]. This initial step is followed by the infiltration of circulating monocytes, neutrophils and T cells [39], which further amplifies the inflammatory response in a stimulated brain. Consistent with these theories, in this study, we demonstrate that microglia were extensively activated in the post-cardiac arrest brain, accompanying exudation of vast myeloid-lineage leukocytes to the brain parenchyma, which suggests that microglial activation and ensuing inflammatory response indeed are critically involved in the pathogenesis of post-cardiac arrest brain injury.

Recent studies have reported that pyroptosis, a ubiquitous form of proinflammatory programmed cell death causing a cascade of inflammatory responses, is implicated in various neurological stimuli [40]. More recently, microglial pyroptosis caused by inflammasome activation and infiltrating leukocytes is shown to be critically involved in the pathophysiology of penetrating traumatic brain injury [13]. Canonical pyroptosis is executed by the cleaved caspase-1, which not only causes cell lysis during pyroptosis but also mediates proteolytic cleavage and release of IL-1β and IL-18 [41]. These IL-1β and IL-18, in turn, propagate the neuroinflammatory response through further activation of resident cells (i.e., microglia) and recruitment of inflammatory cells, such as neutrophils, macrophages, and lymphocytes [40]. Given the over-activation of microglia and the increment in inflammatory cytokines after cardiac arrest as found in this and our previous studies [15, 20, 42–44], we proposed that the overactivated microglia may undergo pyroptosis after cardiac arrest and aggravates the subsequent inflammation in the brain. Consistent with this hypothesis, our data demonstrate that there is indeed intensive pyroptosis and increased caspase-1 activity in the activated microglia population after cardiac arrest, along with elevated levels of IL-1β and IL-18. Moreover, blockage of caspase-1 by a selective inhibitor, Ac-YVAD-cmk, suppresses the pyroptosis, IL-1β and IL-18 levels, as well as inflammatory infiltration but not the number of activated microglia, indicating that the activation of microglia occurs before pyroptosis. Therefore, we provide evidence that microglia are conversed from resting state into activated state after cardiac arrest, followed by the occurrence of pyroptosis and consequential aggravation of sterile inflammation in the brain.

The cleaved caspase-1 comes from autocatalysis and activation of pro-caspase-1 under a platform provided by the assembly of inflammasomes, which are cytosolic protein complexes that contain different structural domains that mediate individual functions [45]. In microglia, the best characterized component is the NLRP3 inflammasome comprised of NLRP3, the adaptor ASC, and pro-caspase-1 [45]. Similar to other NLRs, NLRP3 contains an N-terminal effector binding domain, a nucleotide-binding oligomerization domain, and a C-terminal leucine-rich repeat receptor domain, which bind to ligands and lead to activation of inflammasomes [46]. Most inflammasomes also have ASC as an adaptor molecule, which translocates to the cytoplasm in response to stimuli to form specks and recruit caspase-1 [45]. Under pathological conditions such as amyotrophic lateral sclerosis [47], danger-associated molecular patterns produced by host cells [e.g., ATP, DNA, reactive oxygen species] can initiate the activation of inflammasome, presenting as elevated level and interaction of the components of the inflammasome. Here, we revealed that NLRP3 and ASC were upregulated in the post-cardiac arrest brain with biological interaction, and both NLRP3 and ASC had interaction with caspase-1, which represent the intracellular activation of NLRP3 inflammasome [27]. Besides, NLRP3 and caspase-1 were mainly co-localized in microglia but not neuron, astrocytes, endothelial cells, or oligodendrocyte. Moreover, targeting NLRP3 by MCC950 suppresses the cleavage of caspase-1, elevated levels of IL-1β and IL-18, microglial pyroptosis, as well as infiltration of leucocytes. Therefore, for the first time, we show that the NLRP3 inflammasome-mediated microglial pyroptosis is crucially involved in the development of neuroinflammation after cardiac arrest.

In addition to regulating neuroinflammation, we are more interested in whether the intervention in NLRP3 inflammasome translates into improvements in neurologic outcome and tissue damage. Therefore, we conducted randomized, vehicle-controlled animal studies with blind outcome evaluation to assess the role of MCC950 and Ac-YVAD-cmk in post-cardiac arrest brain injury. MCC950 is a novel, selective small-molecule inhibitor that specifically blocks NLRP3 activation [48], whereby it attenuates inflammation and brain injury after intracerebral hemorrhage and ischemic stroke [49, 50]. Consistent with these findings, we demonstrated that MCC950 showed benefit in improving survival, neurologic function, and lessening neuropathological injuries in post-cardiac arrest rats, indicating that MCC950 may target the NLRP3 inflammasome to prevent post-cardiac arrest brain damage. Ac-YVAD-cmk is an irreversible tetrapeptide inhibitor of caspase-1 with good cell permeability and protects the brain against injury induced by the stimuli such as intracerebral hemorrhage [51]. Here, we add to the current knowledge that the delivery of Ac-YVAD-cmk before cardiac arrest affords neuroprotection against brain injury. It is worth noting that in this study, the two drugs have different effects on improving short-term memory and cognitive function of rats after cardiac arrest, which is reflected in the result that there was no significant difference in the percentage of the time in the target quadrant on probe test after Ac-YVAD-cmk pre-treatment, compared to the vehicle group. In our opinion, the possible reasons for this difference are as follows; firstly, the different routes of administration of the two drugs might have influenced the onset time and efficacy of the drugs. Secondly, we determined the concentrations and doses of the two drugs based on previous studies, which might not be equivalent. To sum up, targeting NLRP3 inflammasome suppresses microglial pyroptosis and neuroinflammation, which further leads to prevention of post-cardiac arrest brain injury.

In this study, we delivered the systemic damage in rats using cardiac arrest modeling, possibly injuring organs other than brain including liver and kidney, whose dysfunction can trigger or aggravate encephalopathy. However, no significant impairment of liver and kidney function was observed in the modeling rats used in our study, and our unpublished data showed no significant changes in creatinine, alanine aminotransferase, glutamate aminotransferase and blood ammonia levels of the post-cardiac arrest animals. Of course, we cannot eliminate that the activation of NLRP3 inflammasome exists in the liver or kidney after cardiac arrest at the molecular level, and pharmacologic blockage of NLRP3 inflammasome may exert a protective effect on these tissues. This should be a limitation of this study.

In summary, we provide evidence that cardiac arrest induces the assembly of NLRP3 inflammasome, which triggers microglial pyroptosis and consequential neuroinflammation and ultimately aggravates brain injury. This work opens up possibilities for further studies to better understand the pathogenesis of post-cardiac arrest brain damage. Furthermore, the demonstration that MCC950 and Ac-YVAD-cmk confer neuroprotection in the model of cardiac arrest advances the current therapies and highlights an under-appreciated effect of these experimental drugs. Although it still requires time to be switched into clinical medication, MCC950 and Ac-YVAD-cmk represent novel and significant strategies to target or address post-cardiac arrest brain injury.

## Conclusion

This study illuminates that microglial pyroptosis mediated by NLRP3 inflammasome is closely related to the severe neuroinflammation after cardiac arrest and critically involved in the pathogenesis of post-cardiac arrest brain injury. In addition, our results provide evidence that MCC950 and Ac-YVAD-cmk could ameliorate neurologic injuries thus helping better outcome, and MCC950 could also improve survival after cardiac arrest, possibly via preventing the activation of NLRP3 inflammasome, the occurrence of microglial pyroptosis, and consequential inflammatory response. Hence, this study provides a new therapeutic strategy.

## Data Availability

The datasets used and/or analyzed during this study are available from the corresponding authors on reasonable request.

## Abbreviations

NLRP3: Nod-like receptor family protein 3
IL-1β: Interleukin 1β
IL-18: Interleukin 18
pro-IL-1β: Precursors of IL-1β
pro-IL-18: Precursors of IL-18
ASC: Apoptosis-associated speck-like proteins containing a caspase recruitment domain
pro-caspase-1: Precursor of caspase-1
GSDMD: Gasdermin D
GSDMD-FL: Full-length GSDMD
pro-caspase-11: Precursor of caspase-11
DMSO: Dimethyl sulfoxide
NDS: Neurologic deficit score
NeuN: Neuronal nuclei
MAP2: Microtubule-associated protein 2
GFAP: Glial fibrillary acidic protein
Iba-1: Ionized calcium-binding adapter molecule-1
Olig2: Oligodendrocyte lineage transcription factor 2
ROSC: Return of spontaneous circulation
ECG: Electrocardiogram
MAP: Mean arterial pressure
HR: Heart rate
CA/CPR: Cardiac arrest and cardiopulmonary resuscitation
ACA: Asphyxial cardiac arrest
qRT-PCR: Quantitative real-time polymerase chain reaction
